# Assessment of Peri-Implant Bone Density Using Intraoral Periapical Radiographs: A Retrospective Observational Clinical Study

**DOI:** 10.3390/healthcare14040541

**Published:** 2026-02-22

**Authors:** Saturnino Marco Lupi, Edoardo Giannini, Viviana Maria Petrantoni, Stefano Storelli, Paolo Boffano, Matteo Brucoli

**Affiliations:** 1 Department of Clinical Surgical, Diagnostic and Pediatric Sciences, University of Pavia, 27100 Pavia, Italyvivianamaria.petrantoni01@universitadipavia.it (V.M.P.); 2 Department of Biomedical Sciences, Surgery and Dentistry, University of Milan, 20122 Milan, Italy; 3 Department of Health Sciences, University of Eastern Piedmont, 28100 Novara, Italy

**Keywords:** dental implants, osseointegration, bone density, alveolar bone loss

## Abstract

**Background/Objectives**: Osseointegration is essential for the long-term success of dental implants, and radiographic assessment may support the evaluation of peri-implant bone healing. This retrospective study evaluated peri-implant radiographic bone density (PIBD) as a potential indicator of osseointegration in patients who underwent successful implant-prosthetic rehabilitation. **Methods**: Patients with at least one endosseous dental implant and a minimum of two standardized periapical radiographs—one at placement (T0) and one during follow-up—were included. Digital radiographs were obtained using the paralleling technique and analyzed with ImageJ^®^. Normalized bone density values were calculated for predefined areas of interest (AOIs). Marginal Bone Level (MBL) changes were also assessed. Statistical analyses included the Shapiro–Wilk test, Kruskal–Wallis test, and Dunn’s post hoc test with Bonferroni correction. **Results**: 88 implants in 64 patients were analyzed (198 radiographs; 1299 AOIs measurements). Normalized bone density showed significant temporal changes in several AOIs, mainly from 3 to 12 months, across coronal/middle/apical regions. PIBD decreased by approximately 8% between T0 and 3 months, followed by a significant increase at one year. MBL values were minimal and well below physiologic thresholds throughout follow-up. No significant correlation was found between MBL and normalized bone density. **Conclusions**: PIBD assessment may be a reliable, non-invasive tool for monitoring osseointegration during follow-up and supporting clinical decision-making in postoperative controls. The temporal pattern observed confirms three radiographic healing phases after implant placement: an initial decrease in PIBD during early remodeling, a subsequent increase reflecting osseointegration, and a final stabilization phase corresponding to tertiary implant stability.

## 1. Introduction

Dental implants are widely recognized as a predictable and effective treatment for oral rehabilitation. However, their long-term success depends on achieving and maintaining proper osseointegration.

The concept of osseointegration was first defined by Brånemark as a direct, structural, and functional connection between living bone and the surface of a load-bearing implant [1,2]. Following implant placement, the surrounding bone tissue undergoes a physiological adaptation characterized by a dynamic equilibrium between osteoclastic resorption and osteoblastic deposition—representing the biological response to functional loading [3,4,5,6,7,8,9]. Between the first and fifth weeks, the newly formed woven bone gradually matures, and after the sixth week it is replaced by lamellar and trabecular bone organized according to implant morphology and mechanical loading conditions. After approximately one month, early bone deposition becomes radiographically evident; at three months, the bone–implant interface shows increased bone density; by six months, cortical bone reaches sufficient thickness; and after one year, bone-to-implant contact typically attains 90–95% [10]. Even after osseointegration is achieved, bone undergoes continuous remodeling cycles driven by biomechanical loading as well as endocrine and metabolic factors such as parathyroid hormone, calcitonin, growth hormone, sex steroids, and vitamin D [3,4,11]. This dynamic equilibrium, known as tertiary implant stability, is histologically characterized by the transformation of cancellous bone into cortical bone [12,13].

Radiographically, increased bone density and mineralization correspond to greater radiopacity around the implant site [14,15]. However if implants are functionally loaded too early, primary failure due to lack of osseointegration may occur [16].

Clinically, identifying when osseointegration has occurred could help in lowering early implant failures and shorten healing time. Even so, longitudinal clinical evidence on PIBD changes assessed on standardized periapical radiographs is still limited, and their relationship with marginal bone level evolution has not been clearly established.

Although histological analyses and CBCT can provide a more detailed assessment of peri-implant bone conditions, they are not routinely applicable in standard clinical follow-up. In contrast, standardized periapical radiographs are commonly acquired during post-operative monitoring, with lower radiation exposure compared to CBCT.

Therefore, the aim of this retrospective study was to evaluate peri-implant radiographic bone density (PIBD) as a potential indicator of bone remodeling during healing, in a cohort of healthy patients who underwent successful implant-prosthetic rehabilitation, using simple and routinely available periapical radiographs.

In contrast to previous investigations relying mainly on CBCT, histology or implant stability measurements, this study offers a longitudinal analysis of normalized PIBD variations in relation to marginal bone level (MBL) using a low-dose imaging approach.

## 2. Materials and Methods

The retrospective observational study was conducted at the Dental School, University of Pavia, and approved by the Unit Internal Review Board (approval ID: 2024-1010). Intraoral radiographs acquired between 1 January 2008, and 31 December 2024, during routine implant follow-up visits were retrospectively analyzed. The primary objective was to evaluate temporal changes in peri-implant radiographic bone density. This retrospective observational study included patients of both sexes, aged over 18 years, who were able to provide valid informed consent. All patients had received at least one endosseous dental implant and had undergone at least two periapical intraoral radiographs—one taken immediately after implant placement (baseline, T0) and one during follow-up. Each follow-up image was assigned to a predefined time interval (T1–T5) according to the elapsed time from placement. Implants placed in both anterior and posterior regions of the maxilla and mandible were included in the analysis (Table 1). All implants were screw-shaped titanium implants with a sandblasted and acid-etched micro-rough surface (diameter 3.3–4.8 mm), placed using a delayed protocol.

### 2.1. Data Collection

**Figure 1:**
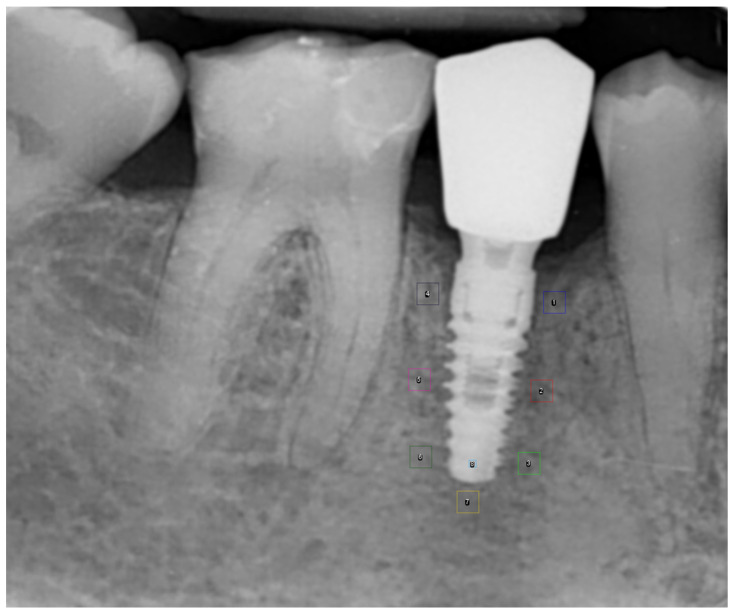
Periapical radiograph showing the position of AOIs from AOI_1 to AOI_8.

Radiographic images were acquired using digital sensors (CEFLA S.C., Size 2, CEFLA S.C., Imola, Italy) and a Fona X70 unit (FONA Italian Radiology, Milano, Italy) operating at 70 kV, with exposure times ranging from 0.20 to 0.32 s. All periapical intraoral radiographs were obtained using the paralleling technique with Rinn film holders to ensure standardized projection geometry, maintaining a tube length of 10 cm and a fixed tube-to-sensor distance of 5.5 cm. Digital image processing was performed using the myray^®^ phosphor scanner (v. 4.31.0.5, CEFLA S.C., Italy). All radiographic images were exported in .jpg format, anonymized, and subsequently processed for analysis. In each radiograph, eight Areas of Interest (AOIs) were defined: seven peri-implant AOIs (AOI_1–AOI_7), each with an area of 5625 pixel^2^, and one AOI located within the fixture (AOI_8) with an area of 625 px^2^. The eighth AOI corresponded to the area of maximum radiographic density, overlapping with the titanium structure of the implant (Table 2, Figure 1).

The AOIs and implant positions were defined on a fixed digital mask from the baseline radiograph, which was subsequently replicated and superimposed on each follow-up image. In cases of minor optical distortion, minimal vertical or horizontal adjustments were applied to ensure precise alignment. Prior to data collection, the examiner underwent specific training and calibration in radiographic assessment to ensure standardized measurement procedures. Radiographic measurements were performed using ImageJ^®^ software, version 1.53k (Wayne Rasband and contributors, National Institutes of Health, Bethesda, MD, USA). All AOIs were placed by a single examiner (E.G.). The collected data were organized in a Microsoft Excel^®^ spreadsheet (Version 2502, Build 16.0.18526.20168) and included: implant ID, age, sex, implant position, time point (T), mesial MBL, distal MBL, AOI identifier, area, group, and gray-scale-based density values (mean, standard deviation, minimum, maximum, integrated density, median, and raw integrated density). For each implant, radiographs were assigned to a specific time point (T) based on the interval elapsed since implant placement (baseline = T0). Six chronological intervals were established, ranging from T0 to T5, as summarized in Table 3.

When AOIs could not be reliably measured due to anatomical overlap or superimposition, the corresponding values were recorded as N.A. and excluded from analysis. AOIs were defined to represent the coronal, middle and apical peri-implant thirds, as well as the apical region. To increase the statistical relevance of the analysis, by enlarging the sample size within each category, AOI measurements were grouped according to their anatomical level (coronal, middle, apical, or apical region) into a new variable, referred to as ROI5, as reported in Table 4. Furthermore, the values recorded for ROI5 were normalized with respect to AOI_8, representing the area of maximum radiographic density, in order to enable accurate comparison across different regions and time points.

Accordingly, variability in PIBD values is expected even among clinically successful implants and should be interpreted, within clinical context, as an expression of local bone remodeling rather than as a direct indicator of implant stability.

Changes in Marginal Bone Level (MBL) were also evaluated across the previously defined time intervals. The MBL was measured mesially and distally to each implant by determining the distance (in millimeters) between two reference points: the implant platform and the most coronal point of bone-to-implant contact. The software was calibrated using the known diameter to eliminate magnification errors. For each pair of mesial and distal MBL values corresponding to the same time interval, the mean MBL was calculated and used for subsequent statistical analysis.

### 2.2. Statistical Analysis

Data were analyzed with R statistical software (version 4.4.2; The R Foundation for Statistical Computing, 2024). Descriptive statistics, including the mean, standard deviation (SD), standard error (SE), and minimum and maximum values, were calculated to summarize the data distribution. The Shapiro–Wilk test was applied to assess data normality. For normally distributed data, one-way analysis of variance (ANOVA) was used to evaluate differences between groups. For non-normally distributed data, the Kruskal–Wallis test was applied for multiple group comparisons. Post hoc pairwise comparisons were performed using Dunn’s test, with Bonferroni correction applied to adjust for Type I error inflation. A *p*-value < 0.05 was considered statistically significant.

## 3. Results

A total of 88 implants placed in 64 patients were analyzed, based on 198 periapical radiographs and 1299 measurements; detailed information on implant number and position is reported in Table 5.

**Figure 2:**
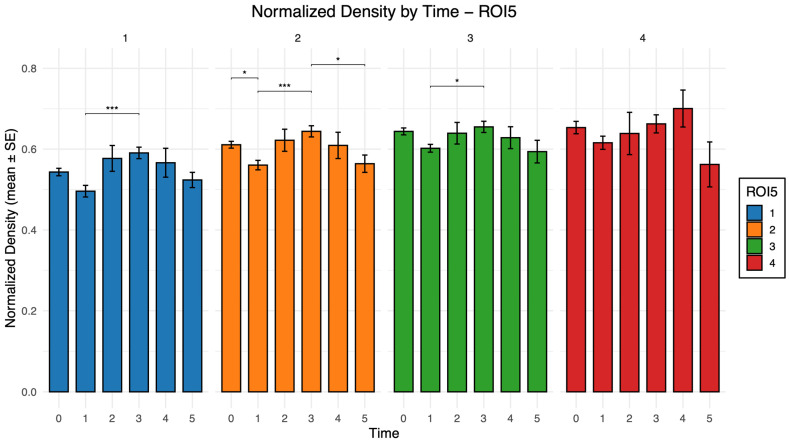
Normalized bone density (mean ± SE) across time intervals (T1: 3 months; T2: 6 months; T3: 12 months) for each ROI5 category. Statistically significant differences (Dunn’s post hoc test) are indicated as * *p* < 0.05 and *** *p* < 0.001.

The normalized bone density values by time and ROI are reported in Table 6 and illustrated in Figure 2.

The distribution of normalized bone density across time and ROI was non-normal; therefore, the Kruskal–Wallis test was applied and yielded a statistically significant result. Post hoc Dunn’s test identified significant pairwise differences, particularly between T1 and T3 (Table 7).

**Figure 3:**
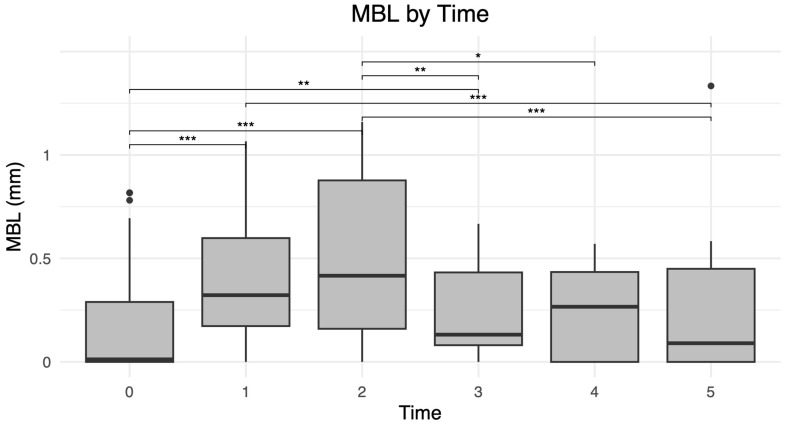
Distribution of marginal bone level (MBL) across the analyzed time intervals (T1: 3 months; T2: 6 months; T3: 12 months). Statistically significant differences (Dunn’s post hoc test) are indicated as * *p* < 0.05, ** *p* < 0.01, and *** *p* < 0.001.

The MBL values by time are reported in Table 8 and illustrated in Figure 3.

The Shapiro–Wilk test for MBL was statistically significant for all ROI5 categories; therefore, the Kruskal–Wallis test was applied and yielded a statistically significant result. The significant pairwise comparisons identified by Dunn’s post hoc test are presented in Table 9. It should be noted that sample sizes decreased at later follow-up intervals, which may limit the robustness of statistical comparisons at these time points.

Furthermore, the potential correlation between MBL and normalized bone density was assessed using Spearman’s rank correlation test; however, no statistically significant association was observed.

## 4. Discussion

In the present study, radiographic PIBD and MBL were evaluated over time and across different AOIs surrounding dental implants. The results provide relevant insights from both clinical and methodological perspectives.

The starting point of this retrospective investigation was the observation of bone architectural and densitometric variations previously reported in experimental animal models [17]. It is important to emphasize that this study analyzed real clinical cases of successful implant-supported rehabilitations, free from peri-implant inflammatory disease. The clinical relevance of this work lies in the possibility of assessing implant health and osseointegration status—and potentially identifying early peri-implant complications—through the analysis of follow-up periapical radiographs routinely acquired during standard patient monitoring.

To enable comparison among different radiographs, PIBD values were normalized to the maximum radiopacity measurable within each image. Normalized bone density showed statistically significant variations over time in specific AOIs, particularly between 3 to 12 months, within the coronal, middle, and apical peri-implant regions. Specifically, PIBD decreased by approximately 8% from baseline to 3 months and then increased significantly at 1 year. A primary consideration relates to the effect size of this finding. Despite statistical significance, the magnitude of the observed variation was relatively modest. This trend reflects the physiological process of osseointegration, characterized by an initial bone resorption phase associated with remodeling, followed by progressive remineralization, which is further influenced by functional loading [5]. From a clinical perspective, monitoring PIBD changes on routine periapical radiographs can contribute to early follow-up strategies, particularly in relation to prosthetic loading time and follow-up planning. In addition, PIBD values were lower in the coronal region. Several factors may explain this finding: (i) some of the radiographs might include implants in the early stages of marginal bone resorption; (ii) image processing filters in the radiographic software may have influenced density readings; (iii) tooth extraction may lead to cortical bone loss and a significant reduction in bone density; and (iv) the natural anatomy of peri-implant bone, which is thinner at the crestal level and thicker in the basal portion [18,19,20,21,22,23,24,25]. Among these hypotheses, since only clinically healthy implants were analyzed and the reduced radiopacity was confined to the coronal region rather than generalized, the first two explanations appear less likely.

MBL was found to be influenced by time, progressively increasing during follow-up, as expected from the literature [21,26]. Marginal bone loss is known to evolve over time and may reflect both physiological remodeling and pathological processes. However, in this study, MBL values were extremely limited and remained well below the threshold of physiologically acceptable levels [26], indicating a stable bone–implant interface, and were also far below clinically relevant thresholds. Therefore, the results should be interpreted within the context of physiological bone adaptation, rather than pathological remodeling. This adaptive process represents a dynamic balance between osteoclastic resorption and osteoblastic deposition, occurring continuously as part of the bone’s response to functional loading [27].

The possible correlation between MBL and normalized bone density was also explored using Spearman’s correlation test, but no significant association was found—neither at the same time points nor with a temporal lag. This suggests that, within the limits of the present dataset, higher local bone density does not necessarily ensure reduced marginal bone loss. The absence of a significant correlation between bone density and MBL is consistent with previous studies, which reported that bone quality is only one of several factors influencing peri-implant bone resorption [28,29,30].

Importantly, the lack of correlation between PIBD and marginal bone loss does not invalidate the observed PIBD trends, as PIBD and MBL reflect different aspects of peri-implant bone changes. PIBD describes density changes related to remodeling, while marginal bone loss represents crestal bone resorption that may occur later or independently.

The limited increase in MBL observed over time, although not correlated with local bone density, may reflect the absence of implants affected by peri-implant pathology in this cohort. It is possible that a marked MBL increase could correspond to a decrease in PIBD in cases of pathological bone loss.

The radiographic bone density measurement method may support the monitoring of peri-implant bone healing; nevertheless, it should be interpreted as an indirect radiographic indicator. Radiographically, three distinct phases of bone healing after implant placement can be identified: (i) an initial decrease in PIBD, corresponding to early bone remodeling; (ii) a subsequent increase in PIBD, reflecting the achievement of osseointegration; and (iii) a stabilization phase, representing the tertiary stability stage.

Recent studies have investigated peri-implant bone changes using advanced radiographic and analytical approaches, including cross-sectional densitometric analyses, fractal-based image evaluation, and three-dimensional imaging combined with biomechanical modeling [31,32,33].

The findings of this study are consistent with previously published reports [12]; however, unlike prior methods—which focused on CBCT-based densitometric analyses, implant stability measures, or baseline bone quality assessments—the present work describes longitudinal peri-implant radiographic bone density changes across standardized follow-up intervals.

The main limitation of the present study lies in its observational and retrospective design, which inherently restricts causal inference. Another limitation concerns the use of two-dimensional digital radiographs to assess PIBD. Although histomorphometric analysis would allow more precise quantification [31], it is not feasible in vivo due to ethical constraints and the retrospective nature of the investigation. Two-dimensional imaging enables density evaluation only at the mesial and distal aspects of the implant. In contrast, a three-dimensional approach such as cone beam computed tomography (CBCT) would allow circumferential assessment of the peri-implant bone [15,29]. However, CBCT is unsuitable for routine follow-up because of its higher invasiveness and potential for metal artifacts that can affect accuracy [32]. Moreover, standardizing MBL evaluation remains technically challenging [33]. and intra-examiner reliability was not formally assessed, which should be considered when interpreting the findings. Radiographic bone density values were normalized to the region of maximum implant radiopacity, which is mainly determined by the constant titanium thickness rather than by bone density variations. The representativeness of the AOIs may also pose a limitation: despite defining eight AOIs, and subsequently grouping them into anatomically defined ROI5 categories, some ROI5 subgroups contained relatively few samples, reducing statistical power; effect size analysis should be considered in future prospective studies. In addition, the exclusive inclusion of clinical successful implants may have introduced a selection bias, as implants affected by failed or compromised osseointegration were not analyzed, limiting predictive conclusions regarding PIBD and implant failure. Another limitation of the present study is the follow-up period, which was limited to two years. Although a longer follow-up period could provide additional information on long-term peri-implant bone dynamics, the primary aim of this study was to investigate the early phases of osseointegration following implant placement, which are most relevant for determining the appropriate timing of subsequent prosthetic procedures. Moreover, no stratified analysis according to implant-related characteristics (e.g., diameter, length, or position) was performed, which should be considered a limitation of the present study.

Finally, not all potential confounding factors—such as smoking status, diabetes, occlusal loading, or prosthetic design—could be fully controlled or consistently recorded, which may have influenced the observed variability.

A prospective study with a longer follow-up period would be valuable to determine whether bone density becomes predictive of implant stability after a certain time or under specific loading or biological conditions. Future analyses should also include failed implants to assess PIBD patterns associated with the loss of osseointegration. Furthermore, the development of dedicated software capable of automatically detecting and quantifying osseointegration status based on radiographic parameters could represent an important advancement in the early diagnosis of peri-implant bone loss.

## 5. Conclusions

This study represents a descriptive, confirmatory analysis of peri-implant radiographic bone density changes observed over time.

The phases of bone resorption and deposition that characterize osseointegration can be effectively identified radiographically at the clinical level through standardized periapical intraoral radiographs, allowing a non-invasive and practical evaluation of peri-implant bone dynamics over time. Nonetheless, higher local bone density does not necessarily ensure reduced marginal bone loss.

The present results are consistent with previous studies, achieved here with a substantially lower radiation dose, supporting the clinical utility of the proposed radiographic method. In the future, routine PIBD monitoring may represent a simple adjunctive tool to support personalized follow-up strategies and clinical decision-making in implant dentistry.

## Figures and Tables

**Table 1:** Inclusion and exclusion criteria.

**Inclusion Criteria**
Patients with at least one osseointegrated dental implant in the anterior or posterior region of either arch.
Availability of high-quality periapical intraoral radiographs acquired using the paralleling technique with Rinn film holders at different follow-up intervals.
Successful implant-prosthetic rehabilitation without clinical or radiographic signs of implant failure.
Good general and oral health at the time of surgery and during follow-up.
Signed informed consent for the use of anonymized radiographic and clinical data for research purposes.
**Exclusion Criteria:**
Systemic diseases affecting bone metabolism (e.g., uncontrolled diabetes mellitus, osteoporosis, metabolic bone disorders) or the use of antiresorptive medications (such as bisphosphonates or denosumab).
History of head or neck radiotherapy or chemotherapy.
Clinical or radiographic evidence of peri-implantitis, peri-implant mucositis, or pathological bone loss unrelated to physiological remodeling.
Poor-quality or non-standardized radiographs (e.g., projection errors, motion artifacts, superimposition, or incorrect angulation).
Implants placed in grafted bone or previously regenerated sites.
Smoking habit exceeding 10 cigarettes per day or history of alcohol/drug abuse.
Missing baseline radiographs (T0).

**Table 2:** Definition of Areas of Interest (AOIs).

AOI	Description
AOI_1	coronal mesial third
AOI_2	middle mesial third
AOI_3	apical mesial third
AOI_4	coronal distal third
AOI_5	middle distal third
AOI_6	apical distal third
AOI_7	apex
AOI_8	area of maximum density

**Table 3:** Study time intervals.

Intervals	Description
T0	Baseline
T1	3 months
T2	6 months
T3	12 months–1 year
T4	18 months
T5	2 years

**Table 4:** ROI5 Variables.

ROI5 Variables	Description
ROI\1	Coronal area of the implant, mesial (AOI_1) and distal (AOI_4); normalized data.
ROI\2	Middle area of the implant, mesial (AOI_2) and distal (AOI_5); normalized data.
ROI\3	Apical area of the implant, mesial (AOI_3) and distal (AOI_6); normalized data.
ROI\4	Sub-apical area (AOI_7); normalized data.
ROI\5	Area of maximum radiopacity used for the calculation of normalized bone density (AOI_8).

**Table 5:** Number and location of implants.

Implant Area	Sample Size	%
Upper molar and premolar	32	36.4
Upper incisor and canine	8	9.1
Lower molar and premolar	41	46.6
Lower incisor and canine	7	8.0
Maxilla	40	45.5
Mandible	48	54.5

**Table 6:** Descriptive statistics of normalized bone density by Time and ROI5.

Time	ROI5	n	Mean	SD	SE	Min	Max
0	1	172	0.543244	0.121315	0.00925	0.180584	0.804884
0	2	171	0.610824	0.1105	0.00845	0.317928	0.830231
0	3	159	0.643863	0.10757	0.008531	0.29089	0.864918
0	4	66	0.653383	0.124404	0.015313	0.326898	0.915092
1	1	65	0.495888	0.115667	0.014347	0.255198	0.781583
1	2	65	0.560458	0.094522	0.011724	0.330075	0.806828
1	3	63	0.602077	0.076759	0.009671	0.436269	0.734019
1	4	26	0.61574	0.082996	0.016277	0.465015	0.745882
2	1	27	0.576977	0.16613	0.031972	0.197098	0.865075
2	2	28	0.621935	0.144619	0.027331	0.259628	0.847885
2	3	24	0.639359	0.13101	0.026742	0.321462	0.816261
2	4	8	0.638722	0.148051	0.052344	0.393089	0.790658
3	1	88	0.590594	0.133496	0.014231	0.270671	0.951417
3	2	88	0.644019	0.129842	0.013841	0.336168	0.976369
3	3	77	0.65505	0.121681	0.013867	0.34465	0.835817
3	4	33	0.662499	0.128738	0.02241	0.369545	0.841303
4	1	16	0.566274	0.143084	0.035771	0.319329	0.815703
4	2	16	0.609218	0.130032	0.032508	0.382663	0.809206
4	3	13	0.628302	0.097673	0.02709	0.46419	0.783047
4	4	6	0.70045	0.112013	0.045729	0.550884	0.857394
5	1	28	0.523627	0.099636	0.018829	0.280105	0.661802
5	2	28	0.5639	0.113503	0.02145	0.351353	0.761587
5	3	24	0.593803	0.137295	0.028025	0.360005	0.860068
5	4	8	0.562114	0.157294	0.055612	0.41746	0.819881

**Table 7:** Significant results of Dunn’s test for normalized bone density.

Comparison	Z	*p* Unadjusted	*p* Adjusted	ROI5
1-3	−4.56998	4.88 × 10^−6^	7.32 × 10^−5^	1
0-1	3.318254	0.000906	0.013587	2
1-3	−4.59342	4.36 × 10^−6^	6.54 × 10^−5^	2
3-5	2.98629	0.002824	0.042358	2
1-3	−3.36076	0.000777	0.011659	3

**Table 8:** Descriptive analysis of MBL by time.

Time	n	Mean	SD	SE	Min	Max
0	364	0.201644	0.329734	0.017283	0	1.754
1	231	0.397591	0.322637	0.021228	0	1.066
2	84	0.520875	0.41984	0.045808	0	1.16
3	105	0.245067	0.219903	0.02146	0	0.667
4	49	0.251786	0.195739	0.027963	0	0.5705
5	77	0.278045	0.392274	0.044704	0	1.334

**Table 9:** Significant pairwise comparisons for MBL (Dunn’s test).

Comparison	Z	*p* Unadjusted	*p* Adjusted
0-1	−8.6846	3.8 × 10^−18^	5.7 × 10^−17^
0-2	−7.64153	2.15 × 10^−14^	3.22 × 10^−13^
0-3	−3.75413	0.000174	0.002609
2-3	3.477874	0.000505	0.007581
2-4	3.00754	0.002634	0.039506
1-5	4.135646	3.54 × 10^−5^	0.000531
2-5	4.681656	2.85 × 10^−6^	4.27 × 10^−5^

## Data Availability

The data supporting the findings of this study are available from the corresponding author upon reasonable request, in accordance with applicable regulations and institutional policies.

## Abbreviations

The following abbreviations are used in this manuscript:

ROI	Region Of Interest
MBL	Marginal Bone Level
PIBD	Peri-implant Radiographic Bone Density
CBCT	Cone Beam Computed Tomography
